# Administering an Appeasing Substance to Gir × Holstein Female Dairy Calves on Pre-Weaning Performance and Disease Incidence

**DOI:** 10.3390/ani10111961

**Published:** 2020-10-24

**Authors:** Beatriz Angeli, Bruno Cappellozza, José Luiz Moraes Vasconcelos, Reinaldo Fernandes Cooke

**Affiliations:** 1 School of Veterinary Medicine and Animal Science, São Paulo State University (UNESP), Botucatu 18618-000, Brazil; biaf.angeli@gmail.com (B.A.); jose.vasconcelos@unesp.br (J.L.M.V.); 2 Nutricorp, Araras 13601-000, Brazil; 3 Department of Animal Science, Texas A&M University, College Station, TX 77845, USA; reinaldocooke@tamu.edu

**Keywords:** bovine appeasing substance, dairy calves, diarrhea, performance, pneumonia

## Abstract

**Simple Summary:**

Stress is a common issue faced by ruminants throughout their productive lives, with significant effects on health and performance of the herd. Hence, strategies that alleviate stress-induced responses with a concomitant increase in performance and health are warranted. Administration of the bovine appeasing substance improved performance and reduced the costs related to pharmacological intervention of pre-weaning dairy calves. Therefore, the utilization of the bovine appeasing substance is a feasible alternative to improve health and performance of the dairy cattle herd.

**Abstract:**

(1) Background: Ruminants often face stressful situations throughout their productive lives. More specifically, pre-weaning dairy calves are exposed to novel environments, feedstuffs, and pathogens that affect their health and performance. Hence, alternatives that reduce stress and promote growth of pre-weaning dairy calves are warranted. Therefore, this study evaluated the effects of biweekly bovine appeasing substance (BAS) administration on performance and disease incidence in dairy Gir × Holstein female calves prior to weaning. (2) Methods: At birth, 140 female Gir × Holstein calves were randomly assigned to receive BAS (SecureCattle; (IRSEA Group, Quartier Salignan, France; *n* = 70) or placebo (BAS carrier, diethylene glycol monoethyl ether; CON; *n* = 70) biweekly until weaning (70 days of age). Calves were allocated into individual housing at random, with no physical contact between treatments to avoid cross-contamination. Experimental treatments (5 mL) were applied topically to the nuchal skin area of each calf. Throughout the experimental period, all animals were observed daily for medical conditions (diarrhea, pneumonia, and others), medical/pharmacological interventions were recorded, and the costs related to these procedures were analyzed. Concurrently with treatment application, calves were individually weighed, and data were analyzed using animal as the experimental unit. (3) Results: Treatment × day and treatment × period (14-day interval) interactions were observed for body weight (BW) and average daily gain (ADG; *p* ≤ 0.05), respectively. Calves receiving BAS had greater BW at weaning (*p* = 0.02) and tended to have a greater BW on day 56 (*p* = 0.06). Similarly, ADG was greater for BAS from days 42 to 56 (*p* = 0.04) and tended to be greater from days 56 to weaning (*p* = 0.10). No differences were observed on the overall occurrence of diseases (*p* = 0.92), whereas the most common observed diseases were diarrhea and pneumonia. The incidence and mean age at which animals were detected with these diseases did not differ (*p* ≥ 0.46). Nonetheless, CON calves detected with disease had a reduced ADG vs. BAS-administered counterparts (*p* < 0.01). No differences were observed between disease-diagnosed BAS vs. healthy CON, but healthy BAS had a greater ADG vs. healthy CON (*p* = 0.03). A treatment effect was observed for the cost per head of each pharmacological intervention (*p* = 0.05). (4) Conclusions: In summary, BAS administration at a 14-day interval improved performance and reduced the costs of pharmacological interventions of pre-weaning Gir × Holstein dairy calves.

## 1. Introduction

Dairy calves are usually exposed to several stressors during the entire pre-weaning period. Among these stressors, abrupt physical separation of the dam after birth, dietary changes, dehorning, grouping, novel environments, and management are the ones that deserve merit [1]. Additionally, between the first and second weeks of life, calves are susceptible to enteric diseases and respiratory syndromes [2] due to a reduced immunocompetence of the still adapting and evolving immune system, whereas previous studies demonstrate that calves with a greater average daily gain (ADG) cope with stress more efficiently compared with counterparts with a reduced ADG [3]. Accordingly, Masmeijer et al. (2019) reported that body weight (BW) influences disease susceptibility of pre-weaned calves [4], and lighter calves are at an increased risk of disease [5]. Hence, it is imperative to improve performance and health of pre-weaned dairy calves by alleviating stress-induced and pathogen-induced responses.

The bovine appeasing substance (BAS) is a technology that has been gaining attention in the livestock industry. The synthetic analogue of the appeasing pheromone is based on a mixture of fatty acids, reproducing the composition of the original substance produced by the dam at calving [6]. In beef cattle, BAS administration improved post-weaning performance and reduced post-weaning mean haptoglobin concentrations [7,8]. Moreover, weekly BAS administration improved milk yield and reduced somatic cell count of dairy cows moved from a confinement to a pasture-based system [9]. To the best of our knowledge, no other study has evaluated the effects of BAS on performance, as well as disease incidence and pharmacological costs of pre-weaning dairy calves. On the basis of this rationale, we hypothesized that BAS administration once every 14-days would improve growth and reduce the occurrence of diseases and treatment costs of pre-weaning dairy calves. Therefore, our objective was to evaluate the effects of BAS administration once every 14-days on performance, disease incidence, and pharmacological costs of pre-weaning Gir × Holstein female dairy calves.

## 2. Materials and Methods

This experiment was conducted at a commercial dairy farm (Fazenda Santa Luzia), located in Passos, Minas Gerais, Brazil (20°43′13″ S, 46°36′36″ W, and elevation of 741 m) from March to May 2020. All animals utilized herein were cared for in accordance with the practices outlined in the Guide for the Care and Use of Agricultural Animals in Agricultural Research and Training [10].

### 2.1. Animals and Treatments

At birth, 140 female Gir × Holstein calves were randomly assigned to receive BAS (IRSEA Group, Quartier Salignan, France; *n* = 70) or placebo (BAS carrier, diethylene glycol monoethyl ether; CON; *n* = 70) every 14 days until weaning at 70 days of age. The BAS active ingredient is based on a proprietary mixture of fatty acids, including palmitic, oleic, and linoleic acids, added at 1% of the excipient, and its effect is estimated to be active in treated animals for 15 days, according to Pageat (1998) [6]. At random (birth body weight (BW) 38 ± 6.4 kg), calves were segregated by treatment into two housing lines (approximately 100 m apart) and allocated into individual housing, with no physical contact between treatments to avoid cross-contamination during the experiment. Experimental treatments (5 mL) were applied topically to the nuchal skin area of each calf, with reference to Cooke et al. (2020), for dose and route of administration [8]. The total number of animals used herein (*n* = 140) was allocated to treatments in 3 consecutive weeks, according to calving, in a manner that kept an equal number of animals enrolled to each treatment within each week.

Immediately after birth, colostrum was individually offered to all animals at a steady volume (L) of 10% of calf’s birth BW, whereas the quality of the colostrum was evaluated immediately at offer to calves and according to methodologies described by Deelen et al. (2014) [11]. From days 1 to 38 of age, all animals received 6 L/day of pasteurized waste milk twice a day (3 L in the morning and 3 L in the afternoon). From days 39 to weaning, animals received 3 L/day of pasteurized waste milk once a day. Calves also individually received a supplement containing (dry matter (DM) basis) 64.0% soybean meal, 30.6% corn meal, and 5.4% mineral–vitamin mix for ad libitum consumption beginning at 5 days of age. Corn silage (33.6% DM, 7.8% crude protein, 63.4% soluble protein (as % crude protein), 32.8% starch, 39.3% neutral detergent fiber, 23.4% acid detergent fiber, 2.9% ether extract, 3.7% lignin, 72.7% total digestible nutrients, 1.85 Mcal/kg of net energy for maintenance, and 1.22 Mcal/kg of net energy for growth) was included into the pre-weaning ration from day 60 to weaning, in order to adapt and start the nutritional transition period from pre- to post-weaning. Additionally, dehorning occurred on day 20 of age, and no medication was used as a standard procedure. On day 70 of age, all animals were weaned and moved to Tifton-85 (*Cynodon dactylon* spp.) paddocks where they received a total mixed ration (TMR) for ad libitum intake.

Throughout the experimental period, all calves had ad libitum access to fresh and clean water and were observed daily for medical conditions by trained technicians, who were blinded to experimental treatments and who followed the standard operational procedures (SOP) of the dairy farm. According to the daily observation, pharmacological procedures required by each individual animal were recorded for further analyses. Additionally, the costs related to these procedures were quantified and analyzed within treatments. These costs were calculated on the basis of the current cost of the medications, the dose of each medication, and by the number of days the animals were treated for such disease. Concurrently with the once every 14-day treatment application (CON or BAS), all calves were weighed, whereas ADG was evaluated at a 14-day interval (periods) and overall from day 0 to 70 of the study (weaning).

### 2.2. Statistical Analysis

All data were analyzed using calf as the experimental unit. Continuous data (BW, ADG, mean age at disease occurrence, as well as days and costs of pharmacological interventions) were analyzed using the PROC MIXED of SAS (SAS Inst., Cary, NC, USA) and the model statement contained the effects of treatment, day (for BW), period (for ADG), or event (for pharmacological intervention), as well as the resultant interactions. Random variables included was animal(treatment), repeated statement was day (for BW) and period (for ADG), animal(treatment) was the subject, and autoregressive compound symmetry was used. Proportion of sick animals by treatment was analyzed using the PROC GLIMMIX of SAS (SAS Inst.) and the model statement contained the effects of treatment, whereas random variables was animal(treatment). All results are reported as least squares means and significance was set at *p* ≤ 0.05, whereas tendencies were denoted if 0.05 < *p* ≤ 0.10.

## 3. Results and Discussion

No treatment differences were observed (*p* ≥ 0.27) on age at first treatment administration (2.8 vs. 2.6 days of age for CON and BAS, respectively; SEM = 0.12) or colostrum quality (7.2 vs. 7.1 g/dL for CON and BAS, respectively; SEM = 0.11). These results demonstrate the similar management newly weaned dairy calves were assigned to and that the colostrum produced by each dam and offered to the calves was in good quality and was not a predisposing factor for any disease observed herein.

**Figure 1:**
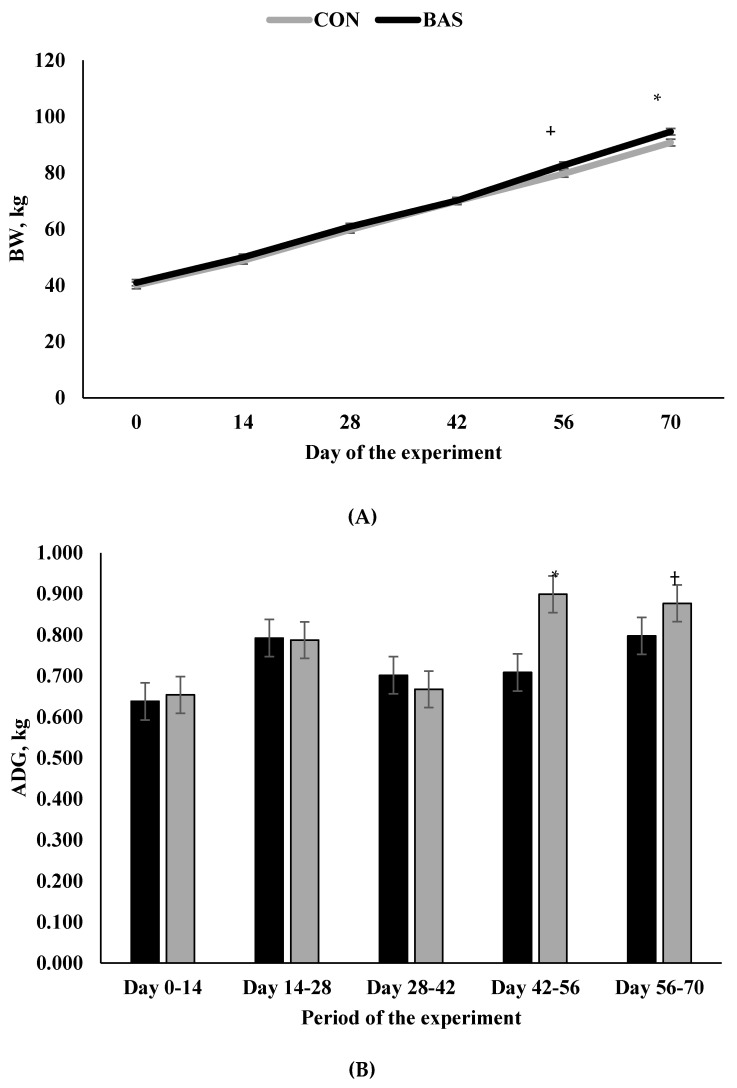
Body weight (BW; **A**) and average daily gain (ADG; **B**) of animals receiving a bovine appeasing substance ((BAS); *n* = 70) or not (CON; *n* = 70) every 14 days during pre-weaning. Treatment × day and treatment × period interactions were observed for body weight (BW) and average daily gain (ADG), respectively (*p* ≤ 0.05). Within day or period: * *p* < 0.05; † *p* < 0.10.

Treatment × day and treatment × period interactions were observed for BW and ADG (*p* ≤ 0.05; Figure 1), respectively. Animals receiving BAS at a 14-day interval had greater BW at weaning (*p* = 0.02) and tended to have a greater BW on day 56 (*p* = 0.06) vs. CON calves (Figure 1A). Similarly, ADG was greater for BAS from days 42 to 56 (*p* = 0.04) and tended to be greater from days 56 to weaning (*p* = 0.10; Figure 1B) when compared with CON (Table 1). These data agree with previous studies in the literature [7,8], in which BAS administration improved growth of newly weaned beef calves.

Three CON calves and two BAS calves died during the experimental period (overall mortality rate = 3.6%). No differences were observed on the overall occurrence of diseases between CON and BAS (*p* = 0.92; 68.0 vs. 70.4%, respectively), whereas the most common diseases that required pharmacological interventions and accounted for 71% of the total occurrences were diarrhea, pneumonia, and calves being diagnosed with both diarrhea and pneumonia. Hence, these diseases were the only ones taken into account for further statistical analysis herein. In agreement with Curtis et al. [12], neonatal diarrhea and pneumonia are well recognized as the leading causes of morbidity, mortality, and antimicrobial use in dairy calves. Per farm SOP, among the pharmacological interventions, saline solution, meloxicam, sulphametoxazol, and florfenicol were administered in diarrhea-diagnosed calves, whereas for pneumonia-diagnosed calves, benzylpenicillin, procaine, dihydrostreptomycin, meloxicam, tulathromycin, enrofloxacin, and bromhexine hydrochloride were available and part of the reported interventions.

The incidence and mean age at which animals were detected with these diseases did not differ between treatments (*p* ≥ 0.46), but the mean age and proportion of each occurrence was different (*p* < 0.01; Table 2). A greater occurrence of diarrhea in the first 3 weeks of calves´ lives was reported, whereas pneumonia was highly reported when calves were 4 weeks old [13]. Nonetheless, Curtis et al. (2016) reported similar ages to those reported herein for diarrhea observation in pre-weaning dairy calves, but with older ages for pneumonia occurrence (44 days of age) [12]. Variations among the present results and others might be due to the environment in which pre-weaned animals were reared, the housing and feeding management, as well as the breed of the dairy cattle herd [12,14].

An additional analysis was performed to evaluate the effects of disease occurrence on ADG of the animals assigned to BAS or CON. A treatment effect was observed (*p* < 0.001), as CON animals detected with any kind of disease had a reduced (*p* < 0.01) ADG when compared with BAS-administered calves, regardless of disease occurrence (0.673 vs. 0.792 and 0.753 kg/day for CON, healthy BAS, and disease-diagnosed BAS, respectively; SEM = 0.021), and tended to have a reduced ADG vs. healthy CON calves (*p* = 0.10; 0.673 vs. 0.718 kg/day, respectively). Moreover, no differences were observed between disease-diagnosed BAS vs. healthy CON animals and disease-diagnosed vs. healthy BAS calves (*p* ≥ 0.20), whereas healthy BAS had a greater ADG vs. healthy CON calves (*p* = 0.03).

During the present experiment, we observed a treatment effect for the cost (USD) per head of each pharmacological intervention (*p* = 0.05; Table 2). The administration of BAS every 14 days to female dairy calves reduced the cost per head of diarrhea (*p* = 0.05) and tended to reduce the cost per head of animals detected with diarrhea + pneumonia (*p* = 0.10), whereas no differences were observed for pneumonia (*p* = 0.65; Table 2). Moreover, number of days required for pharmacological intervention tended to be reduced for BAS- vs. CON-administered animals (*p* = 0.10; Table 2). Considering the benefits of BAS in performance (+3.8 kg of BW) and health (Table 2), its administration every 14 days improved the return on investment of the dairy operation, demonstrating its feasibility in different scenarios. To the best of our knowledge, this is the first dairy cattle data reporting such a result, being concurrent with previous studies from our research group in beef cattle [7,8].

To the best of our knowledge, this is the first experiment evaluating the effects of continuous BAS administration to pre-weaning Gir × Holstein dairy calves. The hypothesis was that administrations once every 14-days would improve health and, consequently, the performance of these animals, as dairy calves face several stressors early in life [1]. Recently, Cooke et al. (2020) reported that a single BAS administration at weaning reduced mean circulating haptoglobin concentration of beef calves 15 days post-weaning, suggesting a better ability of BAS-administered animals to cope with the acute-phase response triggered by a neuroendocrine stressor [8]. The observed delayed response on performance as a result of continuous BAS administration might be related to the disease occurrence observed herein. In other words, it might be speculated that pathogen challenges observed during diarrhea and pneumonia observations might have limited the action of BAS on performance during early periods of the life of the calf but helped to decrease the severity and the amount of pharmacological treatments required to restore calf health. In fact, this rationale would be more appropriate on the basis of the cost of pharmacological intervention per head, which was reduced for BAS-administered vs. CON dairy calves. This is supported by the fact that neuroendocrine stress reactions are often connected with the timing and severity of pathogen challenges, such as the respiratory disease complex in beef animals moving from the farm of origin to a stocker/growing yard [15].

An interesting finding reported herein is that BAS-diseased animals had a similar ADG when compared to healthy CON calves, demonstrating the potential of this strategy to positively impact the performance of animals detected with any kind of illness. Additionally, to the best of our knowledge, no other technology has been shown to positively impact neuroendocrine stress and pathogen-challenged responses. In fact, non-steroidal anti-inflammatory drugs, such as meloxicam, have been shown to improve performance and reduce neuroendocrine-elicited stress responses, but the same immunoprotective effects were not reported upon lipopolysaccharide or vaccination challenge [16]. In agreement, Osella et al. (2018) reported reduced somatic cell count in milk of dairy cows receiving BAS once a week for a 28-day period [9]. Hence, BAS is a technology that might be able to reduce neuroendocrine stress and acute-phase responses [8], as well as hasten the recovery of the body upon pathogen challenges that, in turn, will result in an improved performance post-challenge (stress and/or pathogen).

## 4. Conclusions

In summary, BAS administration at a 14-day interval improved performance and reduced mean duration and costs of pharmacological interventions of pre-weaning dairy calves. Conversely, no treatment effects were observed on disease occurrence. Hence, BAS is a feasible alternative to improve performance, growth development, and alleviate health issues in pre-weaning dairy calves. Nonetheless, more studies are warranted to understand the physiological and immunological mechanisms underlying the responses of ruminants following a disease/pathogen challenge.

## Figures and Tables

**Table 1:** Performance of female Gir × Holstein dairy calves receiving or not receiving (CON; *n* = 70) bovine appeasing substance (BAS; *n* = 70) during pre-weaning.

Item	Treatment		SEM	*p*-Value
	CON	BAS		
Body Weight, kg				
Initial ^1^	40.0	41.0	1.17	0.56
Weaning	90.8	94.6	1.16	0.02
Pre-weaning average daily gain, kg	0.728	0.777	0.206	0.09

^1^ Initial body weight refers to the body weight of animals at first treatment administration and not the birth body weight used for randomization.

**Table 2:** Incidence of diseases, mean age of disease occurrence, days of pharmacological intervention required, and cost per head of pharmacological interventions of female Gir × Holstein female calves receiving (BAS; *n* = 70) or not receiving (CON; *n* = 70) a bovine appeasing substance every 14 days during pre-weaning and diagnosed with any disease during the experimental period ^1^.

Item	Treatment		SEM	*p*-Value		
	CON	BAS		Treatment	Disease	Treatment × Disease
Incidence of diseases, %				0.89	<0.01	0.42
Diarrhea	45.5	52.5	4.95			
Pneumonia	18.8	14.9	2.83			
Diarrhea + pneumonia ^1^	5.9	4.0	1.41			
Others ^2^	29.7	28.7	0.71			
Mean age of disease occurrence, days				0.46	<0.01	0.99
Diarrhea	11.4	10.3	0.96			
Pneumonia	8.4	7.4	1.65			
Diarrhea + pneumonia	4.7	3.0	3.03			
Days of pharmacological intervention, days				0.10	<0.001	0.35
Diarrhea	2.2	1.9	0.25			
Pneumonia	3.3	3.2	0.42			
Diarrhea + pneumonia	4.7	2.9	0.78			
Cost of pharmacological intervention, USD				0.05	<0.001	0.55
Diarrhea	1.91 ^a^	1.11 ^b^	0.283			
Pneumonia	4.24	3.93	0.489			
Diarrhea + pneumonia	6.20 ^a^	4.28 ^b^	0.912			

^a,b^ Different letters denote differences at *p* < 0.10. ^1^ Diarrhea + pneumonia indicate animals that were diagnosed with both occurrences at the same time. ^2^ Other diseases observed include arthritis, hernia, navel infection, anaplasmosis, anemia, neurological issues, and birth weakness.
